# Clinical Holistic Medicine: Holistic Treatment of Rape and Incest Trauma

**DOI:** 10.1100/tsw.2005.38

**Published:** 2005-04-06

**Authors:** Søren Ventegodt, Isack Kandel, Shimshon Neikrug, Joav Merric

**Affiliations:** ^1^ The Quality of Life Research Center, Teglgårdstræde 4-8, DK-1452 Copenhagen K, Denmark; ^2^ Faculty of Social Science, Academic College of Judea and Samaria, Ariel, Israel; ^3^ National Institute of Child Health and Human Development, Faculty of Health Sciences, Ben Gurion University of the Negev, Beer-Sheva, Office of the Medical Director, Division for Mental Retardation, Ministry of Social Affairs, Jerusalem, Israel

**Keywords:** quality of life, QOL, philosophy, human development, holistic medicine, public health, holistic health, holistic process theory, life mission theory, rape, incest, therapy, Denmark

## Abstract

Studies indicate that at least 15% of the female population in western countries has experienced sexual abuse and severe sexual traumas. This paper explains how even serious sexual abuse and trauma can be healed when care and resources encourage the patient to return to the painful life events. When the physician cares and receives the trust of the patient, emotional holding and processing will follow quite naturally. Spontaneous regression seems to be an almost pain-free way of integrating the severe traumas from earlier experiences of rape and incest. This technique is a recommended alternative to classical timeline therapy using therapeutic commands. When traumatized patients distance themselves from their soul (feelings, sexuality, and existential depth), they often lose their energy and enjoyment of life. However, this does not mean that they are lost to life. Although it may seem paradoxical, a severe trauma may be a unique opportunity to regain enjoyment of life. The patient will often be richly rewarded for the extensive work of clearing and sorting out in order to experience a new depth in his or her existence and emotional life, with a new ability to understand life in general and other people in particular. So what may look like a tragedy can be transformed into a unique gift; if the patient gets sufficient support, there is the possibility of healing and learning. Consciousness-based medicine seems to provide severely traumatized patients with the quality of support and care needed for their soul to heal.

## INTRODUCTION

The problem of victimization and revictimization is psychologically extremely complex. Most people believe that the victim is chosen randomly by the offender, but research has shown that victims very often have been victims before and that victimization is often a long chain of life events that contain many different objective events, but are the same mode of victimization.

Research[1,2] has shown that between 33 and 68% of sexually abused victims were subsequently raped. This is compared to an incidence of 17% for nonabused women. Other researchers[3] have found that 18% of repeat rape victims had incest histories, compared to 4% of first-time victims. The research indicated that for many rape victims who have been victimized before, the rape and sexual assault are seldom accidental. These events follow a dark and sad pattern of unconsciously replaying and reliving the role of the victim. This makes the therapy for the rape and incest victim very complex. Most rape victims have earlier incidents of victimization and most incest victims have had difficulties with keeping their boundaries and taking care of their personal safety.

As sexual assaults and rape are among the life events with the most dramatic negative effect on quality of life, the physician must take such traumas extremely seriously. Unfortunately, such sexual assaults are fairly common in the population. Studies from different western countries indicated an incidence of about 15% of girls being assaulted sexually in childhood[1,4,5]. These patients are also more likely to be physically abused by husbands and partners[1,6]. Unfortunately, some are even abused by the therapist who was supposed to heal and protect them[7].

Poor quality of life is statistically connected to bad health. About one in four of the patients seen by the family physician will have such highly painful histories, although most of these sexual traumas remain hidden. The work with these serious problems can, therefore, be a task for specialists. Every physician must be able to handle these traumas when met in the clinic. Fortunately, a loving and caring physician, using the holistic medical toolbox[8,9,10,11,12,13,14,15,16], can help most of the patients to heal, even with serious wounds on the body and soul.

## THERAPY WITH INCEST AND RAPE VICTIMS

Many forms of therapy have proven effective with rape victims, such as cognitive-behavioral therapy[17,18,19], reality therapy[20], and group therapy[21]. Many forms of therapy have also proven effective with incest victims, such as play therapy[22], analytical psychotherapy[23], supportive group therapy[4,8,24,25], couples therapy[26], and family therapy[27], but the results of the therapy is often not completely satisfactory[7,28]. In part, this is because the ethical standards of the therapist working with the incest victims have often been regrettably low. As much as 46% of the incest victims feel abused after the therapy (sexually or otherwise). The toolbox of holistic medicine includes an ethical strategy ("coming from the heart"[29]), which is intended to eliminate the possibility of such malpractice in the holistic medical clinic.

## HOLISTIC TRAUMA TREATMENT: THE USE OF SPONTANEOUS AND GUIDED TIMELINE THERAPY

When we feel that we have lost our value as human beings (as many girls do following a sexual assault) or when we feel that our manhood and self-confidence have been seriously damaged (as many men do following a violent assault), a destructive decrease in self-esteem and self-confidence will result. This is often due to the decisions made during or after the incident to overcome the unbearable feelings of fear, shame, guilt, powerlessness, and hopelessness. Holistic treatment of the after effects of sexual and/or violent traumas is important in order to work on the mind-body dissociation[27], post-traumatic stress, self-blame, sexual dysfunction, and low self-esteem. In this case, holistic treatment is based on classic timeline therapy, going through the incident over and over again, until the patient clearly acknowledges what happened before and can let go of negative decisions made in the heat of the moment.

First, the patient has to feel the pain once again and then everything will be understood. Ultimately, the victim can let go of the life-denying decisions and will feel as though the incident never occurred.

Very often, the whole chain of similar events must be processed to cure the symptoms. Often, this will require thorough and time-consuming work that gives the patients an important learning experience — relief from the painful events and, often, even a gratitude that it happened so that an old, self-destructive pattern finally can be broken.

Aldous Huxley's novel *Island*[30] provides an essential of timeline therapy. Sending the patient back to the trauma can be done by means of the classic timeline commands if the physician has gotten the full trust and acceptance of the patient to receive the necessary holding (awareness, respect, care, acknowledgment, and acceptance[31]):

- Go back to when it happened.
- With your eyes closed, go through the event from the beginning to the end.
- Tell me what happened.

This process should be repeated until the problem has been processed, the learning gained, and the pattern broken. Despite the simplicity of the commands, timeline therapy is not a simple process. Indeed, the skilled timeline therapist must be able to identify the patient's position on the timeline at any time. Also, the experienced therapist rarely needs to apply timeline therapy at all. Meeting and joining the patient exactly where she is will send her back in time *spontaneously*. To be more exact: the patient has never moved beyond the frozen now. So the good doctor should simply join and support the patient with the intention of helping her, then the patient will regress spontaneously or, to be precise, the patient will confront the pain in the frozen now.

In our opinion, therapy with many mental commands is therapy that tries to process things without the requisite emotional holding. In our view, love and compassion constitute a much stronger therapeutic strategy than using power and mental guidance. The former is holistic and practical, the latter keeps within the framework of the mind. From our perspective, Neuro Linguistic Programming (NLP) and mental processes of that kind are not holistic therapy. With love and compassion, holding and processing come quite naturally and, thus, it is holistic healing.

## CLINICAL HOLISTIC MEDICINE

The life mission theory[31,32,33,34,35,36] states that everybody has a purpose of life or huge talent. Happiness comes from living this purpose and succeeding in expressing the core talent in your life. To do this, it is important to develop as a person into what is known as the natural condition. This is a condition where the person knows himself and is able to use all his efforts to achieve what is most important for him. The holistic process theory of healing[37,38,39,40] and the related quality of life theories[41,42,43] state that the return to the natural state of being is possible whenever the person gets the resources needed for existential healing. The resources needed are "holding" in the dimensions of awareness, respect, care, acknowledgment, and acceptance with support and processing in the dimensions of feeling, understanding, and letting go of negative attitudes and beliefs.

The preconditions for the holistic healing to take place are trust and the intention of the healing to take place. Existential healing is not a local healing of any tissue, but a healing of the wholeness of the person, making him much more resourceful, loving, and knowledgeable of himself, his needs, and his wishes. By letting go of negative attitudes and beliefs, the person returns to a more responsible existential position and an improved quality of life. The philosophical change of the person healing is often a change towards preferring difficult problems and challenges instead of avoiding difficulties in life[44,45,46,47,48,49,50,51]. The person who becomes happier and more resourceful often also becomes more healthy, more talented, and more able to function[52,53,54,55].

## ACUTE TRAUMA

> **Female, aged 34 years, with acute trauma:** Arrives in a state of shock and on the brink of tears after having a street fight with her former husband and having had her life threatened by him. He is now on the run from the police, as he has a suspended sentence. She has sent her two children of 5 and 10 years to stay with relatives and friends. Needs psychological assistance, perhaps one weekly session for eight weeks. Sick leave for three weeks. Prescribe urgent counselling – the incident is reviewed four times here, until the patient no longer cries, when confronting it. The psychologist should take over from there.

We refer the patient to a psychologist or a gestalt therapist, but cannot send her home as she is completely emotionally incoherent. We relieve the pressure by means of simple timeline therapy. The patient goes through the incident until the intense emotional reaction has worn off. In this case, the psychologist is also needed because of the social circumstances. Successful trauma therapy is about keeping patients in the present while their attention moves back in time and confronts the traumatic events. Difficult feelings, which the patient receives insufficient support in facing, will allow her to let go of the present and return to the past. Without contact with the present, the patient is technically psychotic and the therapeutic gain from the session will be negligible.

An experienced holistic therapist will notice that the patient is about to lose her mental focus ("third eye closing") before she has left the present. In this situation, we would quickly call in another therapist to support the patient. A patient who is on the brink of psychosis on arrival has to receive ample support, for instance, in the form of a "good father" and a "good mother", before the therapeutic process can begin.

## TRAUMAS IN BODY AND MIND

The classic trauma is a serious and unexpected assault such as rape. In a holistic perspective, even in the case of atrocities, most injuries to the body and mind can heal.

> **Female, aged 16 years, and raped:** In the train, a 16-year-old girl noticed that a young man has taken an interest in her. She avoided his glance, but as she gets off the train on a dark road, he follows her. She becomes scared and tries to run away, but he catches her, throws her to the ground and rapes her. It hurts and she is very frightened. "If you tell anybody, I'll kill you," he whispers to her. She tells no one, but her friends notice that she has become quieter. Once she managed to let go of the sentence "He'll kill me" during therapy, she brightened up and returned to her old self.

This girl has been marked by the incident. The question is why events affect individuals so differently and what actually takes place when we are injured by a trauma. Exactly what was it about the rape that traumatized her? Suffering inflicted on us by the trauma itself, however unpleasant in the present, does not seem to harm us subsequently unless we repress the suffering in the situation and carry it with us. Thus, pain is not traumatizing in itself. Whether or not we become traumatized depends on how we relate to the pain. In the specific situation, the victim can repress the unbearable emotional pain for which she cannot assume responsibility. By drawing a justifying conclusion, she makes the pain go away and that enables her to cope with the situation. But although the pain has disappeared from her conscious mind, it still exists below the surface. After the event, she now carries it along with her. The statement "He'll kill me" is impressed on her subconscious mind and she now has an impression of men that will restrain her in the future, until she relives the pain by being a victim during therapy. In this way, she chooses to suffer without resistance and makes her mind let go of the statement. For lack of a better expression, we call such statements “decisions” — generalized justifications enabling us to disclaim an unbearable responsibility.

## EARLY SEXUAL ABUSE

Early sexual abuse is often extremely traumatizing. Girls (who are most frequently the victims) end up making numerous self-destructive decisions and it is very difficult to become aware of and let go of these decisions. But as the victims address the pain and fully understand the assaults and their nature, they can let go of the negative decisions and life returns. We believe that holistic medicine, when used correctly, can be so effective that no serious scars remain on the soul. The patient can achieve complete recovery, but it takes love and care. Holistic therapy alone is not enough.

> **Female, aged 21 years, and sexually abused:** First quality-of-life (QOL) session: Wants to resolve her inner existential problems that peaked after she had helped a friend recover from a suicide attempt. Has a very difficult personal history, but has tackled it surprisingly well. Has very strong defences, enabling her to appear as a smart and sensitive young woman. SOCIAL: Both parents alcoholics, she lived in a foster home when she was young, was adopted by a couple who divorced four years later, new father also an alcoholic, died when the patient was 9 years old. Subsequently, she lived with the mother of her adoptive father, who ignored her. At the age of 12 years she asked to be placed in a foster home, where she stayed for one year, but the foster family was psychologically mean to her and she felt like a prisoner. Moved to a student hostel on her 18th birthday. On examination: On the couch, however, it can be seen that from the chest down she is practically dead – her abdomen looks more like the abdomen of a corpse, all pale, devoid of blood and life. Strange damage on the skin of both hips, like the cracks in the dermis layer normally seen in pregnant or obese women, but the patient was never overweight. Previous assessment for this, no conclusion. SUBJECTIVE FINDINGS: We talk about emptying the internal waste bin and she appears to be clear and determined about her personal development project: The aim is to find out what you want to do with your life. She wants to provide care, but that is an understandable reaction to her life. Should rather grow up and become independent. She has had about four boyfriends. Her self-esteem needs to be restored. Deserted repeatedly in her life, so she needs to reopen her heart. EXERCISE: Write down your life story – focus on your feelings, thoughts and decisions. Start from the present. What happened? How did you feel? (What decision/conclusion did you make?) What happened? How did you feel? Topics: friendship, love, sex, food, failure – school/work, family, leisure-time. Next appointment in two weeks.
>
> Second QOL session: Has been well, has been very much at home in her abdomen and has felt more than she used to since last session. Has done her homework nine months back. We look at it together. She does not write as much about her feelings, as I (SV) would have liked, it is as though she finds it difficult to recall her feelings. We work on that. EXERCISE: Make friends with your body – do some sport, possibly together with other people, cook some nice meals for yourself, preferably three times a day, explore your sexuality and get to know yourself better, also inside the pelvis and abdomen. EXERCISE: When you continue your autobiography, take the emotional perspective. One hour a day at the most, opens up and then closes. Next appointment in two weeks. Should come sooner if she suddenly feels bad. I think therapy will be hard on her.

Third QOL session: We talk about what theme she is dealing with in her current process. Something about playing dead to survive some horrible situation. Has met a 24-year-old man, whom she has had sex with. The relationship is good. She can feel her emotions. She seems relaxed and happy, and is going camping in the summer and will take our summer course, Life Philosophy that Heals. Should continue the exercises from last session.

Fourth QOL session: Fourth QOL session: Attended the course Life Philosophy that Heals (life purpose: I am wise.) She relived the extensive sexual abuse that she experienced as a child when she was about three years old. Has cried for hours and felt a terrible pain in her reproductive organs and abdomen. Today she feels much more alive and energetic, and she looks much better, although she still has the habit of "playing dead" – she gives, but does not take from her boyfriend, whom I believe she really needs. She has close friends, but she shares only a small part of her life with them. EXERCISE: Rely more on your friends: give and take –take the initiative to be with them, frequently and intimately. Make use of your sexuality. Feeling EXERCISE: Sit on a chair for five minutes every day and sense how you feel. She already does that exercise. How to become truly wise and smart? Write two A4 pages about it. You are going at 1 km/h – it's time to speed up!

Fifth QOL conversation: Things are going well – has set her boundaries with her supervisor, has attained self-respect and her own space. Has experienced close contact with girlfriend. Feels buoyant and happy today. She has reflected sexuality, no problems there, she believes. 1. Rely more on your friends: give and take – and take the initiative to be with them, frequently and intimately – OK, she has done that. 2. Make use of your sexuality– OK about herself. Feeling EXERCISE: Sit on a chair for 5 minutes every day and sense how you feel. She is already doing that - OK. EXERCISE: How to become truly wise and smart? A two-page draft – she has not done that – for next time – write down all sub-aspects you can find of "knowing".

This patient will have to work on herself for years in order to heal the early damage from sexual abuse. Her wounds may not heal completely until the day she finds herself in a warm and genuine relationship.

## DISCUSSION

It is not always possible to work on a certain event in life during therapy. Sometimes, the event is thoroughly repressed, even though well-defined symptoms may have begged the patient to deal with it. Often, the reason for this is that the traumatic event is not a singular event, but occurred as follow-on from earlier traumas and life-denying decisions. Indeed, in our culture, it is common to have experienced a handful or more traumatic events that are related to our problematic themes in life, as mentioned previously. The reason why the individual trauma, which need not be particularly severe, may tip the balance is its contact with earlier, underlying traumas in the particular situation, reactivating their painful content. Most people believe that the anxiety, pain, shame, and hopelessness come from the most recent event. The most recent events have much deeper and more serious roots.

The patient has to reconsider her entire life philosophy and large parts of her personal history in order to regain her balance. The patient needs to be relieved of what may appear, in retrospect, to be a considerable amount of naivety and shallowness. Not until the patient has developed and raised her personal level of responsibility can she integrate the underlying traumas. The patient is now facing two choices: to shut off emotionally and survive, perhaps sustained by symptom-relieving medication such as antidepressants, or to give life a thorough clean up.

With love for our patient comes trust, holding, and processing and this results in holistic healing. Instead of giving commands, giving a surplus of care and resources invites the patient to spontaneously return to the painful events of life. Spontaneous regression seems to be an almost pain-free way of integrating even severe traumas like those that result from rape or incest (affecting at least 15% of the female population[1,4,5]). Interestingly, most of the incest traumas remain hidden in the biomedical clinic, but are often revealed in the holistic clinic, where love or professional care and intimacy is an important part of the therapy.

When traumatized patients distance themselves from their soul, feelings, sexuality, and existential depth, they can easily lose their energy and enjoyment of life. But this does not mean that they are out of the game of life. Although it may seem paradoxical, a severe trauma may be a unique opportunity to gain new understanding and regain participation and full enjoyment of life. The patient will often be richly rewarded for the extensive work of clearing and sorting out and will often experience a new depth in his or her existence and emotional life, with a new ability to understand life in general and other people in particular. So what may look like a tragedy in the beginning of the therapy can be transformed into a unique gift. If the patient gets sufficient support, there is a possibility of healing and learning. Consciousness-based medicine seems to provide severely traumatized patients with the quality of support and care needed for their soul and deepest existence to heal. The most important prerequisite for the healing to happen is the physician's love or care for the patient, and every physician with a loving heart can learn to use the holistic medical toolbox and help his patients to heal existentially.

## ACKNOWLEDGMENTS

This study was supported by grants from IMK Almene Fund. The quality of life research was approved by the Copenhagen Scientific Ethical Committee under number (KF)V.100.2123/91.

## BIOSKETCHES

**Søren Ventegodt, MD,** is the director of the Quality of Life Research Center in Copenhagen, Denmark. He is also responsible for a Research Clinic for Holistic Medicine in Copenhagen and is a popular speaker throughout Scandinavia. He has published numerous scientific or popular articles and a number of books on holistic medicine, quality of life, and quality of working life. His most important scientific contributions are the comprehensive SEQOL questionnaire, the very short QoL5 questionnaire, the integrated QOL theory, the holistic process theory, the life mission theory, and the ongoing Danish Quality of Life Research Survey, 1991–94 in cooperation with the University Hospital of Copenhagen and the late professor of pediatrics, Bengt Zachau-Christiansen, MD, PhD. E-mail: ventegodt@livskvalitet.org. Website: http://www.livskvalitet.org

**Isack Kandel**, **MA, PhD,** is senior lecturer at the Faculty of Social Sciences, Department of Behavioral Sciences, the Academic College of Judea and Samaria, Ariel. During the period 1985–93, he served as the director of the Division for Mental Retardation, Ministry of Social Affairs, Jerusalem, Israel. E-mail: Kandeli@aquanet.co.il

**Shimshon Neikrug, PhD,** is a senior lecturer in the Department of Social Work at College of Judea and Samaria, Ariel in Israel, where he is the director of community studies. He has lectured in Social Work at Tel Aviv University and Bar-Ilan University as well as serving as the director of research in the Bar-Ilan Brookdale Program. Dr. Neikrug has served as consultant to community projects in both the Palestinian Arab sector and Jewish sectors. He is the founder and chair of Yakir-Association for the Third Age. In that capacity, he has directed research projects on quality of life for both private- and public-sector organizations. His present research is on the development of informal support networks for families of children with special needs. E-mail: neikrug@bezeqint.net

**Joav Merrick, MD, DMSc,** is professor of child health and human development affiliated with the Zusman Child Development Center, Division of Pediatrics and Community Health at the Ben Gurion University, Beer-Sheva, Israel; the medical director of the Division for Mental Retardation, Ministry of Social Affairs, Jerusalem; and founder and director of the National Institute of Child Health and Human Development. He has numerous publications in the field of child health and human development, rehabilitation, intellectual disability, disability, health, welfare, abuse, advocacy, quality of life and prevention. Dr. Merrick received the Peter Sabroe Child Award for outstanding work on behalf of Danish Children in 1985 and the International LEGO-Prize ("The Children's Nobel Prize") for an extraordinary contribution towards improvement in child welfare and well being in 1987. E-mail: jmerrick@internet-zahav.net. Website: www.nichd-israel.com
